# Increased sizes and improved qualities of tibia bones by myostatin mutation in Japanese quail

**DOI:** 10.3389/fphys.2022.1085935

**Published:** 2023-01-04

**Authors:** Joonbum Lee, Yuguo Tompkins, Dong-Hwan Kim, Woo Kyun Kim, Kichoon Lee

**Affiliations:** ^1^ Department of Animal Sciences, The Ohio State University, Columbus, OH, United States; ^2^ Department of Poultry Science, University of Georgia, Athens, GA, United States

**Keywords:** myostatin, quail, tibia bone, micro-CT, long bone quality

## Abstract

Production of large amounts of meat within a short growth period from modern broilers provides a huge economic benefit to the poultry industry. However, poor bone qualities of broilers caused by rapid growth are considered as one of the problems in the modern broilers industry. After discovery and investigation of myostatin (MSTN) as an anti-myogenic factor to increase muscle mass by targeted knockout in various animal models, additional positive effects of MSTN mutation on bone qualities have been reported in MSTN knockout mice. Although the same beneficial effects on muscle gain by MSTN mutation have been confirmed in MSTN mutant quail and chickens, bone qualities of the MSTN mutant birds have not been investigated, yet. In this study, tibia bones were collected from MSTN mutant and wild-type (WT) quail at 4 months of age and analyzed by Micro-Computed Tomography scanning to compare size and strength of tibia bone and quality parameters in diaphysis and metaphysis regions. Length, width, cortical thickness, and bone breaking strength of tibia bones in the MSTN mutant group were significantly increased compared to those of the WT group, indicating positive effects of MSTN mutation on tibia bone sizes and strength. Furthermore, bone mineral contents and bone volume of whole diaphysis, diaphyseal cortical bone, whole metaphysis, and metaphyseal trabecular and cortical bones were significantly increased in the MSTN mutant group compared to the WT group, indicating increased mineralization in the overall tibia bone by MSTN mutation. Especially, higher bone mineral density (BMD) of whole diaphysis, higher total surface of whole metaphysis, and higher BMD, trabecular thickness, and total volume of metaphyseal trabecular bones in the MSTN mutant group compared to the WT group suggested improvements in bone qualities and structural soundness of both diaphysis and metaphysis regions with significant changes in trabecular bones by MSTN mutation. Taken together, MSTN can be considered as a potential target to not only increase meat yield, but also to improve bone qualities that can reduce the incidence of leg bone problems for the broiler industry.

## Introduction

In the modern poultry industry, fast growing broilers provide a huge economic benefit by providing a large amount of meat within a short growth period. However, abnormal leg bone development has been considered as one of the major problems of the fast growing broilers (Julian, 2005; Tompkins et al., 2022b; 2022a). Especially, rapid growth of the body and bone during the early growth phase of a broiler is suggested as one of the main causes of poor bone quality in fast growing broilers (Williams et al., 2000, 2004; Tompkins et al., 2022a; 2022b). To minimize economic losses and concerns about animal welfare caused by the leg bone problems of broilers, genetic factors should also be considered and investigated in addition to other aspects including nutritional and environmental factors (Shim et al., 2011; Liu et al., 2015; Huang et al., 2017).

Myostatin (MSTN) is a major anti-regulator in muscle growth and has been investigated in various MSTN mutant animals and humans (Grobet et al., 1997; McPherron et al., 1997; McPherron and Lee, 1997; Schuelke et al., 2004; Mosher et al., 2007; Bi et al., 2016; Lv et al., 2016). Recently developed chickens and quail carrying a mutation in the *MSTN* gene showed increased body and muscle weights, confirming the anti-myogenic function of the MSTN in avian species (Kim et al., 2020; Lee et al., 2020). In addition, a decrease in fat deposition, another major phenotype of MSTN mutant animals (McPherron and Lee, 2002; Ren et al., 2020), was also reported in the MSTN mutant birds (Kim et al., 2020; Lee et al., 2020), indicating the conserved functions of MSTN between mammals and birds.

MSTN knockout mice showed higher bone mineral contents (BMC), bone mineral density (BMD), and bone volume (BV) compared to those of WT mice from Dual-energy X-ray absorptiometry analysis of the whole body at 10 weeks of age (Suh et al., 2020). Also, morphological changes caused by MSTN mutations, such as an extra rib bone and pelvic tilt in MSNT mutant pigs (Qian et al., 2015) and rabbits (Zhang et al., 2019), respectively, were reported. However, the phenotypic changes in bones of the MSTN mutant birds has not been reported, yet.

According to the positive effect of MSTN inactivation on bone qualities demonstrated in mice models (Suh et al., 2020), it is hypothesized that the bone quality of MSTN mutant birds may be improved compared to wild-type (WT) controls. To address this, tibia bones of 4 months old WT and MSTN mutant quail, generated in our previous study (Lee et al., 2020), were analyzed by Micro-Computed Tomography (micro-CT) scanning. Micro-CT is a precise evaluation approach that can provide a comprehensive overview of the architectural characteristics in poultry bones (Tompkins et al., 2022a; 2022b). The three-dimensional structural assessment can provide in-depth understanding behind genetic modulation and bone traits alteration. In addition, breaking strength tests were used to further confirm the quality measured by micro-CT scanning.

## Materials and methods

### Animal care and bone sampling

Animal care protocol and experimental procedures were approved by The Ohio State University Institutional Animal Care and Use Committee (IACUC; Protocol 2019A00000024-R1; Approved 21 January 2022). WT quail and quail carrying homozygous single amino acid deletion mutation in the *MSTN* gene were previously produced using CRISPR/Cas9-mediated genome editing (Lee et al., 2020) and used in this study as WT and MSTN mutant quail, respectively. All quail were maintained together at The Ohio State University Poultry Facility in Columbus, Ohio and fed *ad libitum*. Quail at 4 months of age were euthanized *via* CO_2_ inhalation. After euthanasia, tibia bones of both legs were sampled from eight WT and eight MSTN mutant males. Bones were rolled with paper towels individually and stored in plastic tubes at −20°C freezer until further analysis.

### Analysis of tibia bone

**FIGURE 1:**
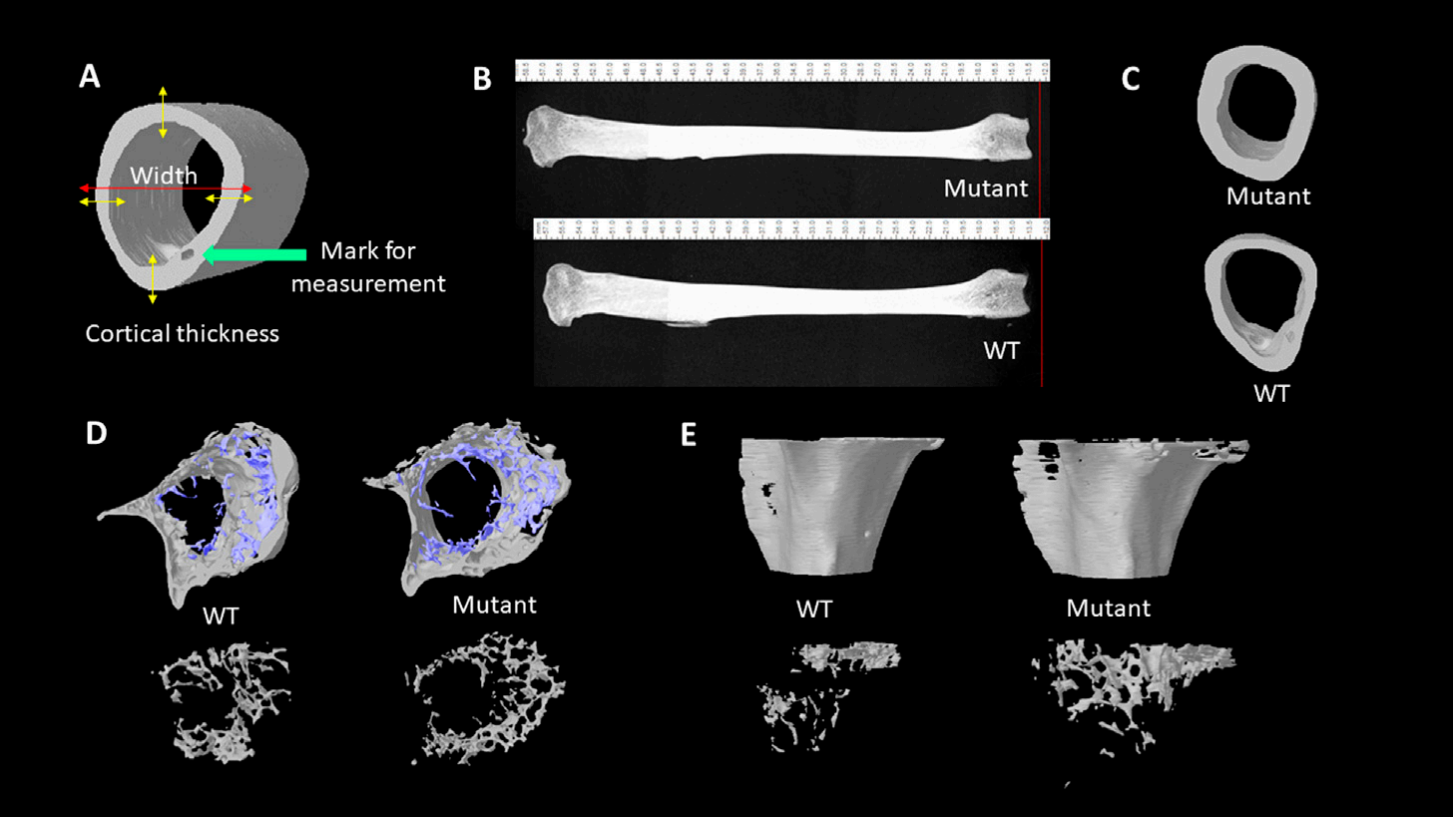
**(A).** Illustration of cortical thickness and wideness measurement. **(B).** Lateral views of 4 months old male quail tibia bones. The length of bones was measured with the CTAn ruler tool. **(C).** Transverse views of tibia diaphysis acquired from the micro-CT scanning. **(D).** Transverse views of tibia metaphysis with trabecular bones (blue) and cortical (grey) structures. Transverse views of tibia metaphysis trabecular bone structures (bottom). **(E).** Lateral views of the reconstructed metaphysis of tibia bones (top). Lateral views of trabecular bones at tibial metaphysis area (bottom).

To evaluate bone morphologic changes and microarchitectural changes, the left tibia bones of eight quail of each group were collected, and muscles were removed before analyzing by micro-CT scanning. Bones scanned according to a standard protocol at 73kV, 136 μA, and a 0.5 mm aluminum filter, and analysis was performed with SkyScan 1172 (SkyScan, Kontich, Belgium). The pixel size was fixed at 25 µm and a 0.25° rotation angle was applied at each step. Images were transferred to CTAn software (CTAn, SkyScan) for 3-D structure construction and quantification. The metaphysis and diaphysis sections of tibia bones were manually selected in the CTAn. Cortical bone and trabecular bone parameters were analyzed, respectively. The following parameters were quantified: BMC, BMD, total tissue volume (TV), BV, bone volume per tissue volume or bone volume fraction (BV/ TV), trabecular thickness (Tb. Th), volume of total pores (Po.V (tot)), total porosity percentage (Po (tot)), tissue surface area (TS), bone surface area (BS), bone surface per total volume or bone surface density (BS/TV), bone surface per bone volume or specific bone surface (BS/BV) (Chen and Kim, 2020). All the bone traits are highly correlated with the health status and bone strength in many species (Chen et al., 2020; Tompkins et al., 2022a; b). Besides, the whole bone length and bone diaphyseal width were measured by using the CTAn ruler tool which measures straight line distance (Figures 1A,B). The large pore for blood vein and nerve fibers to run through (haversian canal) was used as the landmark to select the specific location on the long bone of where the diaphyseal width and the cortical bone thickness were measured using the CTAn software ruler tool (Figure 1A). The cortical bone thickness was calculated by an average thickness of four opposite directional measurements over cortical mid-shaft (Figure 1A).

### Bone breaking strength

Right tibia bones were used to analyze the difference of bone breaking strength (BBS) between WT and MSTN mutant groups. Before measuring BBS, the bones were thawed for 3 h at room temperature and remaining soft tissues around the breaking point of the bones were cleaned. BBS was determined by a three-point bending test using a material testing machine (Stable Micro Systems TA. XT plus 100, Stable Micro System Corp., Surrey, UK) with a 100-kg loading cell and speed of 2 mm/s until the bones fractured.

### Statistical analysis

Student’s *t*-test was used to analyze measurements of left tibia bone sizes and parameters and right tibia bone BBS with the significance level set as *p* < 0.05.

## Results

### The effects of MSTN mutation on tibia bone sizes and strength

The whole tibia bone length was greater in the MSTN mutant group compared to the WT group (Table 1; Figure 1B). Diaphyseal bone width and diaphyseal cortical thickness in the MSTN group were significantly larger than those in the WT group (Table 1; Figure 1C). In addition, BBS of tibia bone was significantly increased in the MSTN group compared to the WT group (Table 1).

**TABLE 1:** Length, width, cortical bone thickness, and bone breaking strength of 4 months old WT and MSTN mutant quail tibia bone.

	Unit	WT	Mutant	Sem	*p*-value
Length	mm	43.104	44.783	0.337	0.007*
Width	mm	2.461	2.546	0.024	0.043*
Cortical thickness^a^	mm	0.287	0.360	0.014	0.006*
Bone breaking strength	KgF	3.633	4.318	0.206	0.005*

^a^ Cortical thickness: a mean thickness of cortical mid-shaft. *means a significantly difference between groups by student’s *t*-test, *p* < 0.05; N = 8 per group.

### The effects of MSTN mutation on tibia bone diaphyseal material and structural properties

The diaphysis is a shaft region in the middle of the tibia bone and mainly formed by a compact outer bone layer, called a cortical bone, and medullary cavity inside (Figure 1C). Using micro-CT scanning, material and structural properties of the whole diaphysis and cortical bone were analyzed (Table 2). Whole diaphyseal structural assay showed higher BMC and BMD of tibia bone in the MSTN mutant group compared to those of the WT group (Table 2). Although the difference of TV was not significant between the two groups, BV of diaphysis in the MSTN mutant group were higher than those in the WT group (Table 2). In addition, BS/BV and BS/TV, but not BS itself, of diaphysis in the MSTN mutant group were significantly different from those in the WT group (Table 2).

**TABLE 2:** Diaphyseal properties of WT and MSTN mutant tibia bone.

		Unit	WT	Mutant	Sem	*p*-value
Whole Diaphysis	BMC	g	13.945	16.258	0.443	0.002*
	BMD	g/mm^3^	0.837	0.922	0.018	0.006*
	TV	mm^3^	16.693	17.659	0.416	0.124
	BV	mm^3^	9.778	10.844	0.237	0.008*
	BS	mm^2^	62.108	60.813	1.261	0.688
	BS/BV	mm^2^/ mm^3^	6.358	5.613	0.148	0.004*
	BS/TV	mm^2^/ mm^3^	3.722	3.450	0.056	0.005*
Diaphysis Cortical	BMC	g	11.006	13.168	0.353	<0.001*
	BMD	g/mm^3^	1.641	1.638	0.030	0.517
	TV	mm^3^	6.738	8.075	0.248	0.003*
	BV	mm^3^	6.670	8.011	0.244	0.003*
	Po.V (tot)	mm^3^	0.074	0.067	0.016	0.106
	Po (tot)	%	0.961	0.912	0.201	0.201

BMC, bone mineral content; BMD, bone mineral density; TV, total tissue volume; BV, bone volume (TV, minus bone marrow volume); BS, bone surface area; BS/TV, bone surface/ total volume; BS/BV, bone surface/ bone volume; Po.V (tot), total volume of pore space; Po. (tot), total pore percentage. *means a significantly difference between groups by student’s *t*-test, *p* < 0.05; N = 8 per group.

As a main component of a diaphysis region, diaphyseal cortical bone in the MSTN mutant group showed higher BMC, TV, and BV compared to those of the WT group (Table 2). However, BMD, Po.V (tot), and Po (tot) of the diaphyseal cortical bone were not significantly different between the two groups (Table 2).

### The effects of MSTN mutation on tibia bone metaphyseal material and structural properties

Unlike diaphysis, trabecular bone is formed inside of the outer cortical bone within the metaphysis region (Figure 1D,E). Therefore, metaphyseal trabecular and cortical bones were separately analyzed along with whole metaphyseal structural assay (Table 3). Whole metaphysis of the tibia bone in the MSTN mutant group showed higher BMC, TV, BV, and TS compared to those of the WT group (Table 3). However, BMD of whole metaphysis was not significantly different between the two groups (Table 3).

**TABLE 3:** Metaphyseal properties of WT and MSTN mutant tibia bone.

		Unit	WT	Mutant	Sem	*p*-value
Whole metaphysis	BMC	g	15.567	19.034	0.758	0.007*
	BMD	g/mm^3^	0.413	0.425	0.011	0.298
	TV	mm^3^	37.81	44.86	1.574	0.007*
	BV	mm^3^	15.543	18.526	0.734	0.017*
	TS	mm^2^	83.531	93.990	2.080	0.005*
Metaphysis Trabecular	BMC	g	0.396	0.899	0.144	0.036*
	BMD	g/mm^3^	0.862	0.898	0.008	0.018*
	Tb.Th	mm	0.079	0.095	0.003	0.023*
	TV	mm^3^	0.450	0.997	0.157	0.037*
Metaphysis Cortical	BMC	g	6.942	8.022	0.204	0.002*
	BMD	g/mm^3^	0.836	0.888	0.024	0.157
	TV	mm^3^	8.370	9.128	0.291	0.104
	BV	mm^3^	7.378	8.429	0.239	0.018*

TS, total bone surface area; Tb.Th, trabecular bone thickness. * means a significantly difference between groups by student’s *t*-test, *p* < 0.05; N = 8 per group.

In the metaphyseal trabecular bone, all the parameters, BMC, BMD, Tb.Th, and TV, were significantly increased in the MSTN mutant group compared to the WT group (Table 3). However, metaphysis cortical bone of the MSTN mutant tibia bone showed higher BMC and BV, but not BMD and TV, compared to those of the WT tibia bone (Table 3).

## Discussion

Avian bones are fast growing, thin, and relatively denser than the mammals (Dumont, 2010). The MSTN mutant quail model has provided a new approach for examining MSTN function regarding to bone quality. As an anti-myogenic regulator, MSTN mutation resulted in increased body weight of quail in our previous study (Lee et al., 2020). In addition to the increased muscle mass, based on the current data, MSTN mutant quail have shown longer and wider tibia bones compared to WT quail, which is similar to MSTN knockout mice having longer femur bones with thicker cortical bones (Hamrick, 2003). Although the BMD remained unchanged in diaphyseal cortical bone between the groups, the cortical thickness and BBS were also increased in the MSTN mutant group compared to the WT group, suggesting that MSTN mutation had increased bone mass and improved bone quality in quail. The diaphysis is the midsection of a long bone that is mainly composed of dense cortical bone (compact bone), where radical growth is characterized at cortical bone over the diaphysis, and the diaphyseal cortical bone structure is essential for structural strength (Isojima and Sims, 2021). In general, cortical bone quality and thickness are highly correlated to BBS (Augat and Schorlemmer, 2006). Thus, the significantly increased BV by *MSTN* gene knockout can result in higher BBS, and the changes in bone mass was the main factor that attribute to better bone quality in the current study.

Like diaphysis results, significant increases in BMC and BV of metaphysis in the MSTN group compared to those in the WT group indicate more mineralization in metaphysis by MSTN mutation. Furthermore, increased TV of whole metaphysis in the MSTN mutant group compared to the WT group also indicates wider metaphysis in the MSTN mutant group. At metaphysis, a higher BMC, BMD, Tb.Th, and TV of the metaphyseal trabecular bone were observed in the MSTN mutant group compared to the WT group, suggesting improved trabecular bone quality by MSTN mutation. Trabecular bone is a dynamic structure compared to cortical bone. Trabecular bone quality is one of the major contributors of bone strength and quality (Garrison et al., 2011). The changed structure and decreased bone mineral content/density were reported in pathogenic challenge models in broilers (Raehtz et al., 2018; Tompkins et al., 2022b). Therefore, the improved trabecular bone quality can possibly increase bone integrity to confronting stressed condition. Interestingly, unlike higher BMD of whole diaphysis in the MSTN mutant group, BMD of whole metaphysis was similar between the two groups. This might be further supported by the finding that a 22% increase of BMC of the whole metaphysis bone results mainly from a similar increasing rate, 19%, of TV of whole metaphysis bone rather than changes in BMD in the MSTN mutant group.

Similar to the results from structural assay of tibia bones of MSTN mutant quail, diaphyseal cortical bone thickness and BV, without BMD improvement, were higher in tibia and humerus bones of MSTN mutant mice compared to those of WT mice (Suh et al., 2020). In addition, trabecular bone quality was improved in quail by MSTN mutation as shown in higher trabecular BMC in the humerus bone (Hamrick et al., 2002) and trabecular BMD in fifth lumbar vertebra (Hamrick et al., 2003). These results indicate a similar function of the *MSTN* gene on regulation of bone development and quality, along with anti-myogenic function, between mammals and birds. In murine *in vitro* studies, a direct effect of the MSTN on bone remodeling status by promoting osteoclast differentiation (Dankbar et al., 2015) and inhibiting osteoblast differentiation (Qin et al., 2017) was reported. MSTN mutation can positively affect bone formation rate and reduce bone resorption, eventually resulting in a larger bone volume in MSTN knockout mice (Suh et al., 2020). Osteoblasts contribute to bone formation and mineral deposition, while osteoclasts resolve the bone and initiate the bone remodeling (Matsuo and Irie, 2008). The differentiation and activity of these two bone-related cells are critical in maintaining bone quality and integrity (Alliston, 2014). Studies using mice *in vitro* cell models indicated that MSTN interacts with regulators essential in bone homeostasis by regulating osteoblast and osteoclast activity (Ewendt et al., 2021). For example, studies using mice osteoblastic MC3T3 cells show that the increased level of MSTN promotes expression of bone growth inhibitor such as sclerostin (SOST), and also the osteoclastogenic stimulators such as RANKL (Qin et al., 2017). Besides, the increased MSTN also associated with a marked reduction of Runx2, which is one of the essential osteoblastogenic regulators, *via* the down-regulation of the WNT signaling (Qin et al., 2017). With the current research, it is likely that the activity of osteogenic differentiation or osteoclastogenic differentiation can be mediated by MSTN mutation, which can positively affect bone formation rate and remodeling status, eventually resulting in a larger bone volume in MSTN mutant quail. Moreover, in mice studies, the increased bone mass was associated with the change in the osteogenic differentiation of osteoblast, and MSNT was shown to inhibit adipogenesis. It is well-known that the WNT pathway is one of the most important signaling pathway to promote osteoblast differentiation, and the activation of this pathway showed an anti-adipogenesis and anti-chondrogenesis (but promotes chondrocyte hypertrophy) function during bone formation (Olivares-Navarrete et al., 2011; Lee et al., 2017; Riddle and Clemens, 2017). The activation of WNT pathway can block PPAR-gamma-induced adipogenesis and induce RUNX2 expression, which commit mesenchymal stem cells differentiated into the osteoblast phenotype (Santos et al., 2010). Thus, the regulation of WNT pathway, and the interaction between osteogenic differentiation and adipogenic differentiation can be critical in understanding MSTN in bone homeostasis in future studies.

Chicken muscle yield has been considered one of the most important traits in genetic breed selection (Bailey et al., 2020). However, the continuous selection for rapid growth and high muscle yield has shifted the broilers’ center of gravity and altered the biomechanical structure (Huang et al., 2019). The unbalanced development between muscle and skeleton has been unexpectedly associated with the incidence of metabolic and skeletal disorders in modern broiler breeds. For broilers, most of skeleton disorders occur on the long bone (tibia and femur) (Cook, 2000; Akyüz and Onbaşılar, 2020). Thus, leg disorders are a significant cause of welfare issues in broilers. Compared to slow growing chickens, fast growing broilers is characterized with a relative lower mineral contents, higher porosity, and lower BBS (Williams et al., 2000). Significantly increased mineralization in the tibia bone by MSTN mutation as shown in higher BMC and BV in all diaphyseal and metaphyseal regions suggests MSTN as a potential genetic factor that can improve bone mineralization, bone strength, and structural integrity in fast growing broilers.

In summary, MSTN mutation resulted in longer, wider, thicker, and stronger tibia bone with significant improvements of bone quality parameters in both diaphysis and metaphysis regions of quail tibia bone. The current study provides scientific evidence for potential applications of MSTN to not only increase meat yield, but also improve bone quality in poultry.

## Data Availability

The original contributions presented in the study are included in the article/supplementary material, further inquiries can be directed to the corresponding authors.
